# Factors influencing change in walking ability in patients with heart failure undergoing exercise-based cardiac rehabilitation

**DOI:** 10.1016/j.ijcard.2018.05.021

**Published:** 2018-10-01

**Authors:** Natasha Sutherland, Alexander Harrison, Patrick Doherty

**Affiliations:** Department of Health Sciences, University of York, York, UK

**Keywords:** Exercise, Rehabilitation, Heart failure, Walking ability, Physical capacity, Secondary prevention

## Abstract

**Objectives:**

Exercise-based cardiac rehabilitation (CR) is an effective intervention for patients with heart failure (HF), in which one of the main targets is to increase physical capacity. In the HF population this is traditionally assessed using distance covered during a walking test. This study aims to establish the extent to which change in walking ability, in HF patients attending CR, is determined by patient characteristics and service provision.

**Methods:**

The study utilised routine clinical data from the National Audit of Cardiac Rehabilitation to perform a robust analysis. Change, in metres, between pre- and post-CR six-minute walk tests was calculated. Multivariate linear regression models were used to explore the relationship between patient characteristics, service-level variables, and change in metres walked.

**Results:**

Complete and valid data from 633 patients was analysed, and a mean change of 51.30 m was calculated. Female gender (−34.13 m, *p* = 0.007), being retired (−36.41 m, *p* = 0.001) and being married/in a relationship (−32.54 m, *p* = 0.023) were all significant negative predictors of change. There was an additional negative relationship with body mass index (BMI) whereby for every unit increase in BMI, predicted change reduces by 2.48 m (*p* = 0.006).

**Conclusions:**

This study identified significant patient-level characteristics strongly associated with limited improvement in walking ability following CR. Improving physical capacity is a core component of CR, therefore services should aim to account for baseline characteristics identified in this study as part of tailoring the CR intervention around the individual. Pre- and post-CR physical capacity assessments, which constitute minimum standards for CR, are worryingly low and should be given high priority.

## Highlights

• Heart failure patients make mean change of 51 m on post-rehab six-minute walk test.
• Being female, in a relationship, retired & higher BMI predict reduced change.
• We should account for baseline characteristics when tailoring rehab to the patient.
• Higher priority should be given to pre- & post-rehab physical capacity assessments.

## 1 Introduction

Cardiac Rehabilitation (CR) is a clinically effective intervention for patients with Heart Failure (HF) with published clinical guidance for implementation of an exercise-based rehabilitation programme [[1], [2], [3]]. A summary of clinical trials on exercise-based CR in HF confirms a reduced risk of overall and HF-specific hospitalisation with clinically important improvement in the quality of life [1]. However, despite extensive evidence for CR, eligible patients are missing out on CR with fewer than 20% of HF patients accessing such services in the UK. Across Europe the number of programmes offering CR to patients with HF is increasing and in the UK over 90% of programmes have a CR referral process for patients with HF [4]. Data from the UK National Heart Failure Audit suggests that 7% to 20% of patients with a diagnosis of HF are referred to CR from either general wards or cardiology wards with wide variation in referrals between hospitals. Survival analysis of patients with HF, based on referral to CR, demonstrated improvements of 12% compared to patients not referred to CR [5].

One of the key targets for CR programmes is to increase the physical capacity of patients, which is best achieved through tailored and supervised exercise training [1,6]. In order to quantify the benefits of exercise training for patients, numerous filed tests of walking capacity have emerged which allow clinicians and patients evaluate change in walking capacity. One of the most popular walk tests for patients with low capacity is the six-minute walk test (6MWT) which requires patients to continuously walk at their own preferred pace, until they are either unable to continue, or the maximum time of 6 min is met. At the end of the test, the distance walked is measured in metres, and recorded [7].

Studies have tried to develop expectations around meaningful clinically important difference (MCID) which has led to estimates varying between 14 and 32 m, depending on the underlying pathology [[8], [9], [10]]. There is a lack of clarity about which factors influence walk test distance, and uncertainty around their interpretation when using the 6MWT. This is important to clarify as these factors may help explain why so few patients start CR without undergoing an exercise test.

The study aim is to establish the extent to which change in walking ability, in patients HF attending CR, is determined by clinical service and patient characteristics.

## 2 Methods

This cohort study was reporting following the Strengthening the Reporting of Observational Studies in Epidemiology (STROBE) guidelines [11].

### 2.1 Six-minute walk test

In this study physical capacity is operationalised as walking ability. Patients perform this test once at the start of the exercise programme, and repeat it on completion of CR.

### 2.2 Data

The source of data is the National Audit for Cardiac Rehabilitation (NACR) database which is created through routine anonymised data entry from 226 cardiac rehabilitation centres across England, Wales and Northern Island. The data is entered by CR professionals approved by their local Caldicott Guardian, and the data is hosted by the NHS Digital. The data collection is under 251 exemption which allows collection of data without consent, however patients can opt out. The NACR captures the entire pathway of CR, from presenting event in hospital through to rehabilitation in the community. The analysis included all cases where a diagnosis of HF led to a new referral to cardiac rehabilitation between January 2013 and December 2016. Cases were deemed eligible where both a pre- and post-rehab 6MWT had been completed.

### 2.3 Outcome measure

The study explores predictors of change, in metres, between the pre- and post-CR 6MWT. The change was calculated by subtracting the first six-minute walk test result from the second. As there is no agreement on the MCID for the 6MWT, we have opted to measure the outcome linearly, in metres, which will also allow us to quantify the strength of this relationship [9].

### 2.4 Predictors

Age, gender, ethnicity, height, BMI, smoking status, baseline fitness, diabetes, depression, blood pressure, cholesterol and number of comorbidities were included as known predictors of fitness/physical performance. In addition, duration of CR; time between referral and invite to CR; and participation in early rehabilitation (e.g. whilst still an inpatient) were included as service-level factors that have also been shown to influence performance. Finally, marital status; employment status; waist measurement; angina; arthritis; asthma; COPD; anxiety; having a psychologist and/or physiotherapist employed at the CR centre; and the number of hours a psychologist and/or physiotherapist is contracted to work per week were included as additional hypothesized factors influencing performance.

The presence of diabetes, angina, arthritis, asthma and COPD were ascertained from the patient's medical history. Abnormally high scores on the Hospital Anxiety and Depression Scale (HADS) were used to identify patients with anxiety and/or depression as this was thought to be a more accurate indicator of the patient's mental health status at the time of CR.

### 2.5 Statistical analysis

The analysis explored initial associations between variables and the outcome, change. Independent *t*-test and one-way ANOVA were used to compare the means of the categorical variables. Pearson's rank correlation coefficient was used to investigate association of the continuous variables.

This initial analysis gave an indication of which variables may or may not yield a significant relationship, however all variables were then entered into a multiple stepwise backward linear regression model to investigate if these relationships were upheld in multivariate analysis. All models were tested for fitting assumptions [12].

The structure of the four steps of the model is summarised in Table 1. Predictors' inclusion in the final model were based on 95% confidence intervals and a *p* value of <0.05.

**Table 1:** Structure of stepwise regression model: predictors of change on six-minute walk test post-CR.

1. Socio-demographic factors	2. Cardiac risk factors	3. Patient medical status	4. Service level factors
Age	Smoking status	Number of comorbidities	Duration of CR
Gender	Diabetes	Angina	Time between referral & invitation to CR
Ethnicity	Baseline walking ability	Arthritis	Undertook early CR
BMI	Systolic blood pressure	Asthma	Psychologist employed
Height	Diastolic blood pressure	COPD	Psychologist contracted hours
Waist size		Anxiety	Physiotherapist employed
Marital status		Depression	Physiotherapist contracted hours
Employment status			

CR: cardiac rehabilitation.

## 3 Results

**Fig. 1:**
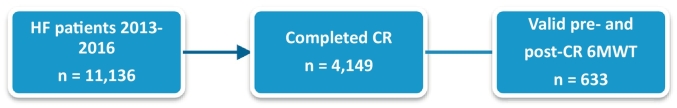
Study flow and sample size. HF: heart failure; CR: cardiac rehabilitation; 6MWT: six-minute walk test.

The study population is summarised in Fig. 1 with 633 patients included in analysis. The mean change between pre- and post-CR 6MWT was 51.30 m.

Table 2 summarises the predictor variables which yielded a significant *p* value. Table 3 summarises the final results of backward stepwise linear regression. The final model included 97 patients. Smoking status was the only variable that was omitted from the original model as descriptive statistics revealed that only 5% of the population identified as current smokers, which was too small a number for the regression model to operate.

**Table 2:** Predictors of improvement on 6MWT with significant *p* values from independent *t*-test and correlation coefficient.

Variable		Mean change in metres (SD)/correlation coefficient	*p* value
Achieving 150 min exercise per week	Yes	40.28 (75.19)	0.024
	No	57.13 (70.72)	
Baseline performance on pre-CR 6MWT		−0.322	<0.001
Received early rehab	Yes	66.44 (65.74)	0.001
	No	45.82 (70.90)	
Psychologist employed	Yes	81.29 (69.24)	0.017
	No	49.05 (69.24)	
Psychologist contracted hours		0.151	0.009

6MWT: six-minute walk test; CR: cardiac rehabilitation; SD: standard deviation.

**Table 3:** Predictor variables for change in distance walked between two 6MWT.

Variable	Unstandardised coefficient (b)	95% CI	*p* value
Gender (female)	−34.13	−58.80 to −9.46	0.007
BMI	−2.48	−4.23 to −0.72	0.006
Retired	−36.41	−57.92 to −14.90	0.001
Married/relationship	−32.54	−60.50 to −4.57	0.023
6MWT at baseline	−0.26	−0.35 to −0.16	<0.001
Psychologist hours	1.83	0.07 to 3.58	0.041
Constant	263.50	186.11 to 340.89	<0.001

6MWT: six-minute walk test; BMI: body mass index; CI: confidence interval.

Female gender, raised BMI, being retired, being in a relationship, and having a higher baseline performance were all significant negative predictors of change. The contracted number of hours of the centre psychologist was found to be a significant positive predictor. Importantly none of the comorbidities data or the number of comorbidities featured as a statistical determinant of walk test outcome.

The largest effect size that was observed is being retired, which predicted a change of 36.41 m less than those who were employed. This is closely followed by female gender, predicted to achieve 34.13 m less of a change than males; and those currently married or in a relationship predicted to achieve 32.54 m less than those who identify as single. There is a negative linear relationship between BMI and change, such that for every unit increase in BMI, a patient is predicted to achieve 2.48 m less. A similar relationship exists with baseline performance, whereby for every metre achieved at baseline, a patient will achieve 0.26 m less on the follow up 6MWT. However, the number of hours a psychologist is contracted with the CR programme creates a positive linear relationship whereby for every hour a psychologist is employed, a patient will achieve a further 1.83 m of change.

## 4 Discussion

This is the first study which investigates factors influencing walking performance in HF patients using multivariate analysis. It is of importance due to the need to be able to appropriately interpret the results of walking tests in this population, especially with increasing numbers of HF patients now accessing CR. This study revealed that having a psychologist involved in CR delivery had a significant positive influence on the patients' change in walking ability, with a positive correlation between the psychologist's contracted number of hours, and the size of the change. This relationship is already noted in patients with traditional CR conditions, where psychological interventions have not only been found to alleviate psychological symptoms [13] but have also been linked to reduced event recurrence and even mortality [14,15]. The significance of this relationship in HF patients adds strength to the argument for psychological input throughout all CR programmes.

Conversely, female gender has a significant negative impact on outcome, with a large effect size of −34.13 m. It is established that women have much lower adherence to CR programmes than men [16] with lack of social support and high burden of family responsibilities identified as some of the barriers women face [17]. However it is troubling that this might be having such a detrimental effect on their outcomes. With poor adherence, and poor outcomes, there is an argument for CR tailored to the specific needs of female participants. It has been suggested that incentive-based strategies and/or increased availability of home-based CR programmes could improve female participation [17].

As somewhat a surprise, retirement was found to significantly limit a patient's opportunity to improve their walking ability; even when controlled for age. We hypothesized that the age categories were too narrow to be meaningful in our sample, causing retirement status to act as a surrogate for older age. The regression model was repeated, with age in a binary format of under 65, or 65 and over (to align with the UK retirement age). Even when controlled for age in this way, the regression still produced retirement as a significant negative predictor, meaning there is something about this status, irrespective of age, which influences patients' change in walking ability. This may be related to an overall decline in physical activity during the transition into retirement [18] though additional research would need to look into this further.

It was also surprising to find that being married or in a relationship also had a significant negative impact on patients' change in walking ability, as this is usually understood to be a protective factor in cardiovascular disease [19] and also associated with better CR attendance [20]. A possible explanation could be a lack of motivation in this group, especially if their partner does not have an active lifestyle themselves, contributing to a home environment that is resistant to lifestyle change, and potentially reducing the impact of CR. Again, research would need to be done to further elucidate this connection.

Finally, increasing BMI had significant negative correlation with the patients' change in walking ability. Patients with a raised BMI may have a worse baseline performance, but it would then be expected that they would have a greater scope for improvement. It is therefore curious to note that as BMI increases, the change in walking ability decreases. A reduced adherence to CR in obese patients has previously been observed, [21] although the evidence is nowhere near as compelling as it was for female gender. It is possible that patients with higher BMI may need either a more prolonged exercise programme, or an additional intervention to reduce BMI before they can see a meaningful improvement in walking ability.

The coefficients of the regression model are of particular interest in the context of MCID. Although there is no consensus on an exact figure, the literature so far suggests it could be anywhere between 14 and 32 m [[8], [9], [10]]. The coefficients for female gender, retirement and marriage/relationship are all greater than this value, which means that by having just one of these characteristics, a patient is much less likely to achieve the MCID.

Perhaps one of the most meaningful findings from this study was that neither number, nor type of comorbidity was found to have a significant impact on the change in walking ability. Fig. 1 showed that only 633 of the 4149 HF patients completing CR had performed both a pre-and post- 6MWT. There is concern that this low assessment rate is possibly due to clinicians' worries over the safety of this test in lower physical capacity patients. However, this study indicates that the 6MWT is a feasible test to perform in multi-morbid patients, and that comorbidities should not be used as exclusion criteria from performing the 6MWT at pre- and post-CR assessments of HF patients.

## 5 Limitations

This study was limited by its small sample size, *which is unlikely to be representative of all eligible patients. Our analysis of patient demographics such as age; gender; and comorbidity; between the study group and overall HF registrants found no significant difference between the populations.* There are presently very few HF patients participating in CR, which limits the amount of data available. We felt there is a need to analyse and share these initial findings to help clinical teams feel reassured that patients can perform and benefit from CR. *The statistical methods used in this paper were justified and assumptions for the regression met, however, repeat analysis of this study using a greater sample size will help to validate these findings.*

## 6 Conclusion

This study found that female gender; retirement; being married or in a relationship; and increasing BMI are significant negative predictors of change in walking ability. These characteristics ought to be taken into account in order to provide a CR service tailored to the individual, giving them a better opportunity to achieve a meaningful improvement. Conversely a positive relationship between a psychologist's involvement in the CR programme and change in walking ability was also found, which adds further support to the call for psychologists as compulsory members of the CR multidisciplinary team.

The 6MWT is a feasible test of walking ability in routine practice, even in multi-morbid patients, and its use should be encouraged in HF and low capacity patients undergoing CR.

## Funding

This research project was supported by the British Heart Foundation through a research grant awarded to the University of York (R1680901).

## Conflict of interest

The authors report no relationships that could be construed as a conflict of interest.
